# Percutaneous Cryotherapy and Radiofrequency Ablation of Renal Masses: Multicenter Comparative Analysis with Minimum 3-Year Follow-up

**DOI:** 10.1590/S1677-5538.IBJU.2024.0565

**Published:** 2025-01-13

**Authors:** Umberto Carbonara, Francesco Ditonno, Alp T. Beksac, Ithaar Derweesh, Clara Cerrato, Antonio Celia, Giovanni Costa, Lorenzo Bianchi, Jeffrey Elbich, Brandon Wilson, Lance J. Hampton, Savio D. Pandolfo, Giuseppe Basile, Fernando J. Kim, Riccardo Schiavina, Umberto Capitanio, Jihad Kaouk, Riccardo Autorino

**Affiliations:** 1 Santa Maria Hospital Department of Urology Bari Italy Department of Urology, Santa Maria Hospital, Bari, Italy; 2 Rush University Medical Center Department of Urology Chicago IL USA Department of Urology, Rush University Medical Center, Chicago, IL, USA; 3 University of Verona AOUI Verona Department of Urology Verona Italy Department of Urology, AOUI Verona, University of Verona, Verona, Italy; 4 Glickman Urological and Kidney Institute Cleveland Clinic Cleveland OH USA Glickman Urological and Kidney Institute, Cleveland Clinic, Cleveland, OH, USA; 5 University of California San Diego School of Medicine Department of Urology La Jolla CA USA Department of Urology, University of California San Diego School of Medicine, La Jolla, CA, USA; 6 San Bassiano Hospital Department of Urology Bassano Del Grappa Italy Department of Urology, San Bassiano Hospital, Bassano Del Grappa, Italy; 7 University of Bologna Department of Urology Bologna Italy Department of Urology, University of Bologna, Bologna, Italy; 8 Vascular Interventional Radiology VCU Health Department of Radiology Richmond VA USA Department of Radiology, Vascular Interventional Radiology, VCU Health, Richmond, VA, USA; 9 Division of Urology VCU Health Richmond VA USA Division of Urology, VCU Health, Richmond, VA, USA; 10 University of L’Aquila Department of Urology L’Aquila Italy Department of Urology, University of L’Aquila, L’Aquila, Italy; 11 IRCCS San Raffaele Hospital Division of Experimental Oncology/Unit of Urology Milan Italy Division of Experimental Oncology/Unit of Urology; IRCCS San Raffaele Hospital, Milan, Italy; 12 Denver Health Division of Urology Denver CO USA Division of Urology, Denver Health, Denver, CO, USA

**Keywords:** Kidney Neoplasms, Cryotherapy, Radiofrequency Ablation, Treatment Outcome

## Abstract

**Background:**

Different modalities of percutaneous thermal ablation (PTA) have been used as possible minimally invasive nephron-sparing treatments for small renal masses (SRMs). The present study aimed to compare long-term outcomes of two guidelines-recommended ablative techniques, cryotherapy (CRYO) and radiofrequency ablation (RFA).

**Materials and Methods:**

Data of patients with single cT1 solid renal mass undergoing CRYO or RFA between 2004 and 2020 were retrospectively retrieved from a multi-institutional international database. Oncologic outcomes included "technical success", local recurrence-free survival (RFS), distant metastasis-free survival (MFS), and overall survival (OS). Intraoperative and postoperative complications, length of stay (LOS), and re-admission rate within 30 days were registered. Major complications were defined as CD grade ≥III. Baseline features and treatment outcomes were analyzed using descriptive statistics. RFS, MFS, and OS were estimated using the Kaplan-Meier method.

**Results:**

Overall, 643 patients were included, of which 492 (71.2%) underwent CRYO, and 151 (21.8%) RFA, with a median follow-up of 43 and 37 months, respectively (p=0.07). Technical success was achieved in 96.5% of CRYO vs 93.4% of RFA (p=0.09). No difference in terms of overall (CRYO: 10.4% vs RFA: 6%; p=0.1) and "major" (CRYO: 0.8% vs RFA: 1.3; p=0.06) post-operative complications were observed. RFS (CRYO:85.7%; RFA:84.9%, p=0.2), MFS (CRYO: 96.9%; RFA: 95.8%, p=0.4) and OS (CRYO: 89%; RFA: 87.4%; p=0.8) were comparable.

**Conclusions:**

CRYO and RFA are both valid minimally invasive options for the treatment of small renal tumors. They are particularly suitable for patients who are not good surgical candidates as they offer very low risk of major procedure-related complications. For the right indication, they both offer favorable mid to long term oncologic outcomes.

## INTRODUCTION

Different thermal ablation (TA) approaches have been developed as minimally invasive nephron-sparing surgery for small renal masses (SRMs). Among the others, percutaneous cryotherapy (CRYO) and radiofrequency ablation (RFA) are widely used techniques for treating SRMs through a minimally invasive, nephron-sparing approach. CRYO involves the application of extreme cold to induce cellular damage and tumor cell death, while RFA relies on thermal energy generated by radiofrequency waves to achieve tumor necrosis (1). Both procedures are performed under imaging guidance, typically using computed tomography or ultrasound, with CRYO providing enhanced visualization through the formation of an ‘ice ball’ around the treated area. These techniques are especially valued for preserving renal function, reducing hospital stay, and decreasing complications compared to partial nephrectomy (PN), particularly in patients who are poor surgical candidates (2).

Indeed, according to American Urological Association (AUA) guidelines, CRYO and/or RFA should be considered as options for patients with SRMs less than 3 cm. Great emphasis on the need to discuss the higher risk of tumor recurrence and the potential need for re-treatment during patient counseling (3). European Association of Urology (EAU) adopts a more cautious position, reserving percutaneous TA (PTA) to frail and/or comorbid patients, due to the existing uncertainties regarding its clinical effectiveness (4). Such a discrepancy is mainly because current evidence on PTA approaches is predominantly based on single-center and population-based retrospective studies (5-7).

We hypothesized that CRYO and RFA would yield comparable long-term oncologic outcomes in patients with SRMs. Therefore, the aim of the present study is to describe and compare the long-term outcomes of these two guidelines-recommended PTA procedures in a large international multicenter cohort.

## MATERIALS AND METHODS

### Study design and population

Data were retrieved from a multi-institutional international database including patients undergoing PTA in seven U.S. and European centers between 2004 and 2020. Inclusion criteria were adult patients (18 years) with single cT1 solid renal mass who had undergone either CRYO or RFA. Exclusion criteria included multifocal or metastatic renal cell carcinoma at presentation, incomplete follow-up data or missing data in outcomes of interest, and lack of post-procedural imaging confirming ablation outcomes. As an analysis of deidentified data, the study obtained exempt status after being reviewed by the local Ethics Committee. Data sharing across participating centers was obtained.

Baseline characteristics, together with clinical, treatment, and post-treatment data were collected. Baseline patient features included demographic data, body mass index (BMI), American Society of Anesthesiologists (ASA) score, history of smoking, diabetes mellitus and hypertension, preoperative estimated glomerular filtration rate (eGFR) calculated by using the Chronic Kidney Disease Epidemiology Collaboration formula (8), and medical history of chronic kidney disease (CKD) ≥ class III. Clinical staging included tumor size, tumor staging according to TNM status, RENAL nephrometry score (9), hilar location, and tumor biopsy.

Treatment details and outcomes included intraoperative and postoperative complications overall, and ≤30 days major according to Clavien-Dindo [CD] classification (10), length of stay (LOS), and re-admission rate within 30 days. Complications with CD grade ≥ III were defined as "major complications".

Oncologic outcomes included: "technical success", defined as the extension of ablation defect beyond tumor margin with the absence of residual enhancement in the ablation bed on imaging obtained immediately after the procedure (11), local recurrence-free survival (RFS), defined as a new focal enhancement in the ablation bed or enlargement of the ablation defect on follow-up imaging, distant metastasis-free survival (MFS), as extrarenal disease on imaging, with or without pathologic confirmation, and overall survival (OS), as death by any cause.

A trifecta composite outcome was evaluated for each treatment, including: "technical success", as a surrogate for oncological outcome; absence of major perioperative complications, as a proxy for surgical outcome; <10% reduction in eGFR at 90 days, as a surrogate for functional outcome. A trifecta outcome as surrogate of overall treatment quality was considered achieved only if all three above conditions were satisfied.

### Statistical analysis

Statistical analysis was conducted according to guidelines (12). Patients were stratified into two groups according to treatment modalities. Means and standard deviations (SD) or median and interquartile range (IQR) were adopted to report normally distributed and non-normally distributed continuous variables, respectively. Proportion and frequencies were used to report categorical variables. Patient demographic characteristics and treatment outcomes of each cohort were analyzed using descriptive statistics, as appropriate.

Local RFS, distant MFS, and OS were estimated using the Kaplan-Meier method. The follow-up duration for RFS and MFS was determined from the treatment to recurrence and/or metastasis, respectively. For OS, the follow-up duration was calculated from treatment to the last follow-up visit. Patients with benign histology at pre-treatment biopsy were censored for the assessment of oncological outcomes. To identify significant predictors of "trifecta" achievement, we conducted logistic regression analysis adjusting for age, BMI, ASA score, RENAL Nephrometry Score, and procedure type.

All statistical tests were performed with SPSS^®^ 25.0 (IBM Corp. Armonk, NY, USA) and statistical significance was set at p<0.05.

## RESULTS

### Baseline characteristics

Table-1 summarizes demographics and tumor characteristics. Overall, 643 patients who underwent PTA were included in the analysis. Of these, 492 (71%) and 151 (29%) were treated with CRYO and RFA with a median follow-up of 43 and 37 months, respectively (p=0.07).

**Table 1:** Demographics and tumor characteristics.

		Overall	CRYO	RFA	p
Patients, n (%)		643	492 (71)	151 (29)	-
Female Gender, n (%)		200 (29.2)	155 (31.5)	45 (29.8)	0.693
**ASA score, n (%)**					0.9
	1	7 (0.9)	6 (1.2)	1 (0.7)	
	2	156 (24.2)	118 (24.0)	38 (25.2)	
	3	406 (63.2)	309 (62.8)	97 (64.2)	
	4	74 (11.6)	59 (12.0)	15 (9.9)	
Age, years, mean (SD)		68.8 (10.7)	68.5 (10.7)	69.8 (10.6)	0.181
BMI, kg/m^2^, median (IQR)		27 (24-30)	27 (26-32)	27 (26-32)	0.8
CKD ≥ III stage, n (%)		170 (26.4)	118 (23.9)	52 (34.4)	**0.011**
Diabetes history, n (%)		166 (25.8)	132 (26.9)	34 (22.5)	0.278
Preop. eGFR, mL/min, median (IQR)		65.4 (63-66)	67.0 (63-71)	62.5 (58-66)	**0.015**
Clinical size, cm, median (IQR)		2.5 (1.5-2.5)	2.5 (1.5-2.5)	2.5 (1.5-2.5)	0.4
**Clinical T stage, n (%)**					0.1
	T1a	604 (93.9)	462 (93.9)	142 (95.3)	
	T1b	37 (6.1)	30 (6.1)	7 (4.7)	
RENAL score, median		6 (4-6)	6 (4-6)	6 (4-6)	0.6
Malignant, n (%)		500 (77.8)	384 (78.1)	116 (76.8)	0.5
Clear cell		204 (29.6)	169 (34.4)	35 (23.2)	
Papillary		96 (14.0)	73 (14.8)	23 (15.2)	
Chromophobe		26 (3.8)	22 (4.5)	4 (2.7)	
Other/Unspecified		174 (25.3)	120 (24.1)	54 (35.8)	
Benign histology, n (%)		94 (14.6)	69 (14.0)	25 (16.6)	
Oncocytoma		48 (6.9)	23 (4.6)	24 (15.8)	
Angiomyolipoma		4 (0.6)	4 (0.8)	0	
Others		43 (-)	42 (-)	1 (-)	
No Biopsy/Data not available, n (%)		49 (7.6)	39 (7.9)	10 (6.6)	

No differences in terms of mean age (p=0.1), ASA score (p=0.9), median BMI (p=0.8) were observed between the two cohorts. Also, tumor features like median clinical tumor size (p=0.4), rate of cT stage (p=0.1), and RENAL nephrometry score (p=0.6), were comparable for both RFA and CRYO. The RFA group had a lower median baseline eGFR of (62.5 vs 67.0 mL/min; p=0.015) and a higher rate of CKD ≥ III stage (34.4 vs 23.9%; p=0.011).

At preoperative biopsy, 77.8% of the whole cohort presented malignant histology and 7.6% of the patients did not have data on the biopsy. among which the most common subtype was clear cell RCC at 29.6%.

### Treatment outcomes

Treatment outcomes are described in Table-2. Imaging-based "technical success" was achieved in 95.8% of the whole cohort, with no difference between the approaches (CRYO: 96.5% vs RFA: 93.4%; p=0.09). A significantly higher number of intraoperative complications was observed during CRYO (3.3% vs 0%, p=0.02). No difference in overall (CRYO: 10.4% vs RFA: 6%; p=0.1) and "major" (CRYO: 0.8% vs RFA: 1.3; p=0.06) postoperative complications were reported.

**Table 2:** Treatment and oncological outcomes.

		Overall (n=643)	CRYO (n=492)	RFA (n=151)	P value
Technical success*, n (%)		616 (95.8)	475 (96.5)	141 (93.4)	0.09
Intraoperative complications, n (%)		11 (1.7)	11 (3.3)	0	0.02
Overall postop. complication, n (%)		60 (9.3)	51 (10.4)	9 (6.0)	0.1
Major postop. complications, n (%)		6 (0.9)	4 (0.8)	2 (1.3)	0.06
Hospital stays, days, median (IQR)		2 (1-3)	2 (1-3)	2 (1-3)	0.3
Last follow-up, months, median (IQR)		41.5 (39-42)	43 (42-44)	37 (35-39)	0.07
30-day readmission, n (%)		4 (0.8)	3 (0.6)	1 (0.6)	0.5
Local recurrence, n (%)		96/536 (17.9)	65/417 (15.6)	27/119 (22.7)	0.07
Time to recurrence, months, median (IQR)		12 (10-14)	12 (10-14)	11 (10-13)	0.4
Distant metastasis, n (%)		24/536 (4.5)	17/417 (4.1)	7/119 (4.6)	0.4
Time to distant metastasis, months, median (IQR)		23 (22-25)	17 (15-19)	26 (24-28)	0.26
**Deaths, n (%)**					
	Overall	114/536	80/417	34/119	0.3
	Cancer-related	(21.3) 11/536 (2)	(19.2) 8/417 (1.9)	(28.6 3/119 (2.5)	0.7
Time to Death, months, median (IQR)		31 (28-34)	40 (37-43)	39 (38-40)	0.1

* Treatment and oncological outcomes of patients undergoing cryotherapy and radiofrequency ablation

Overall, 94 (14.6%) patients who had a benign histology report were excluded from the analysis of oncological outcomes, as well as patients without an oncologic follow-up. Therefore, oncological outcomes were evaluated in 536 patients, including 417 patients treated with CRYO and 119 with RFA.

Within the overall cohort, local recurrence was observed in 96 (17.9%) patients. Of these, 65 (15.6%) and 27 (22.7%) patients after CRYO and RFA, respectively.

**Figure 1:**
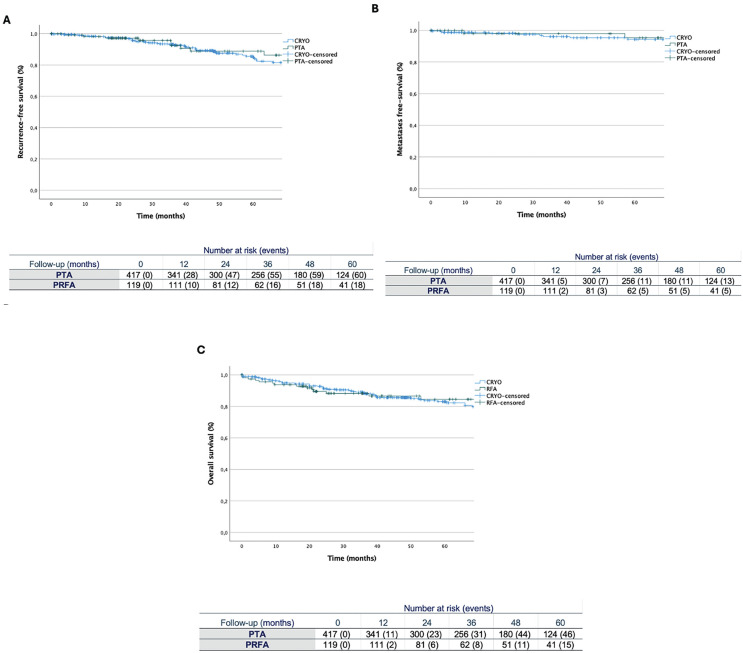
Kaplan–Meier curves of oncological outcomes for patients undergoing percutaneous thermal ablation: A) recurrence-free survival; B) metastasis-free survival; C) overall survival.

After 5 years, local RFS rates were 85.7% for CRYO and 84.9% for RFA, with 124 and 41 patients still at risk, respectively. There was not a statistically significant difference in local RFS between different subgroups (p=0.2) (Figure-1A).

Distant metastasis developed in 24 (4.5%) patients with a median onset time of 23 months. The 5-year MFS rates were 96.9% for CRYO and 95.8% for RFA without any difference (p=0.4) (Figure-1B). Overall, 114 patients died over an average of 31 months after PTA treatment. The 5-year OS rates for CRYO and RFA were 89% and 87.4%, respectively (p=0.8) (Figure-1C). At 5 years, there were 124 patients still at risk in the CRYO group, and 41 in the RFA group.

Trifecta outcome was achieved by 496 patients (76.3%), of which 324 (77.7%) received CRYO, and 85 (71.4%) underwent RFA, with no statistically significant difference between the two groups (p=0.3). According to logistic regression analysis, both BMI (odds ratio [OR] 1.08, 95%CI 1.02-1.15) and RENAL score ≥7 (OR 1.21, 95%CI 1.08-1.57) was related to a decreased likelihood of trifecta achievement.

## DISCUSSION

To the best of our knowledge, this study represents among the few to compare mid to long-term oncological outcomes of CRYO and RFA in a large multicenter setting. Our findings corroborate the existing evidence which is mostly based on single-center case series (Table-3).

**Table 3:** Review of the available literature on percutaneous thermal ablation.

Author	Year	Design	Technique	Patients	Outcomes*				Follow-up (months)
					RFS	CSS	OS	Success rate	
Tracy, et al. (16)	2010	Single center Single arm Retrospective	RFA	215	93%		85%	97%	27
Marshall, et al. (17)	2020	Single center Single arm Retrospective	RFA	100	92%		75%	^100%	62.8
Kim, et al. (39)	2013	Single center Single arm Retrospective	CRYO	124	85%	100%	85%	87%	30.2
Knox, et al. (18)	2020	Single center Single arm Retrospective	CRYO	277				95.6%	27.4
Bhagavatula, et al. (40)	2020	Single center Single arm Retrospective	CRYO	307	RFS: 88% Local RFS: 95%	99%	76%		41
Stacul, et al. (14)	2021	Multicenter Single arm Retrospective	CRYO	338	82.4%		91%	95.9%	26.9
Andrews, et al. (25)	2019	Single center Comparative Retrospective	PN vs CRYO/RFA	1422	PN: 97.7% RFA: 95.9%, CRYO: 95.9% p>0.05	PN: 99.3% RFA: 95.6% CRYO: 100% p>0.05	PN: 92% RFA: 72% CRYO: 77% p<0.05		6.3-9.4 years
Millan, et al. (24)	2022	Multicenter Comparative Retrospective	PN vs CRYO/RFA	2276	PN: 97.4% PTA: 88.1% p<0.05		PN: 99% PTA: 97.4% p=0.9		24-28.8

* Review of the available literature on percutaneous thermal ablation

Analyzing patients’ baseline characteristics, our groups showed comparable features. Looking at those that can potentially influence the PTA outcomes such as BMI (13), tumor position (14), and complexity of the renal mass (15) we found no differences in the two groups. This mitigates potential selection bias and confounding factors, especially analyzing the oncological outcomes.

No significant difference between CRYO and RFA was observed in terms of oncological outcomes. Image-based technical success was achieved in 96.5% of patients after CRYO and 93.4% after RFA, without difference (p=0.09). Concerning RFA, early results by Tracy et al. indicated a technical success rate of 97% after the primary procedure, with a mean follow-up of 27 months (16). More recent data with a longer follow-up at 62.8 months showed a success rate of 90% after RFA treatment (17). As for CRYO, our success rate aligns with those reported in the literature. Indeed, two single-arm retrospective studies assessed a success rate of 95% (14, 18). Our comparative analysis reaffirms these promising results of percutaneous TA, extending them to a large multi-institutional setting (19). It can be argued that the use of computed tomography during the percutaneous approach offers a more precise visualization of the ice ball for CRYO and facilitates treatment monitoring for both modalities, in contrast to the use of ultrasound in laparoscopic technique (20). Indeed, intraoperative ultrasound guidance can play a valuable role in developing a tailored surgical approach during kidney surgery (21).

When we look at time-to-event outcomes, our cohort shows encouraging results, without significant differences between the two groups. RFS rates were 85.7% and 84.9% at 5 years after CRYO and RFA, respectively (p=0.2). MFS rates of 96.9% for CRYO, and 95.8% for RFA were reported (p=0.4). Moreover, OS was 89% and 87.4% for CRYO and RFA, respectively (p=0.8). While these outcomes are in line with those previously reported in literature (22), evidence comparing ablative treatments to PN remains inconclusive (23). Millan et al. directly compared PN to PTA, revealing a significantly higher 2-year local or distant RFS for the former (97.4% vs 88.1%, p=0.003) (24). However, the relatively short follow-up period and potential sample size discrepancies after propensity-score matching may account for these conflicting results. Conversely, Andrews et al. reported no significant differences in local RFS (PN: 97.7%, RFA: 95.9%, CRYO: 95.9%, all p>0.05), and distant MFS (PN: 98%, RFA: 93%, CRYO: 100%, all p>0.05) for cT1a renal masses, with a longer follow-up (25). In our prior experience with PN compared to PTA, we observed similar outcomes, with local recurrence occurring in 4% vs. 6.7% (p=0.3) and the onset of metastasis in 6% vs. 7.5% (p=0.4) of patients, respectively. Moreover, a superior safety profile for PTA emerged as evidenced by lower postoperative complication rates and better preservation of renal function (26). This may confer an additional advantage to PTA over PN, particularly in more fragile patients (27).

Our findings revealed a low rate of postoperative overall (9.3%), and major complications (0.9%) in the overall cohort, with no significant difference between the two techniques. Interestingly, CRYO showed significantly higher intraoperative complications when compared to RFA (3.3% vs. 0%, p=0.02). However, the clinical significance of this result is uncertain. The overall percentage of intraoperative complications remains low, and consistent with those of previous studies (28, 29). The low incidence of intra- and postoperative complications may speculate an advantage of the percutaneous approach when compared to laparoscopic procedures, as higher complication rates have been previously reported in studies on following laparoscopy TA (30). For this reason, AUA recommends a percutaneous approach when ablation is considered as a therapeutic option (3).

Another paramount outcome of nephron-sparing surgery is the preservation of renal function (31). According to a retrospective analysis by Woldu et al., PTA techniques allowed better preservation of renal parenchyma, especially when compared to PN. In their analysis, the authors observed that the kind of surgery was the strongest predictor of renal parenchyma volume preservation (32). In a multicenter comparative analysis of trifecta outcomes, we reported a significant worsening of postoperative renal function 1 year after PN, compared to PTA (33). Nevertheless, some other studies did not identify any significant differences between PN and CRYO (34) or RFA (35), making it difficult to draw conclusions on this subject. However, potential reasons for poorer parenchymal preservation after PN include the greater complexity of treated renal masses, as well as the vascular clamping and the tension created by renorrhaphy, which may contribute to additional tissue loss (32). Therefore, a comprehensive evaluation of oncological, surgical, and functional outcomes becomes pivotal when counseling patients on potential treatment options, to provide patient-tailored solutions, especially when a nephron-sparing treatment is mandatory.

We evaluated the efficacy of these techniques using a surrogate of surgical success as the trifecta, which has been extensively reported for PN (36). However, it is not routinely used for PTA studies. The trifecta can help authors compare different studies and techniques. In our cohort, CRYO and RFA appeared comparable in trifecta achievement rates. Our analysis suggested that BMI and a higher RENAL score could adversely affect trifecta achievement. Similar findings were observed in the trifecta analysis of patients undergoing PN, where these same variables, among others, were inversely related to trifecta achievement (37). Indeed, this composite outcome corroborates the overall success quality of these procedures and the comparability of their long-term results.

This study provides novel insights into the long-term efficacy of CRYO and RFA as nephron-sparing treatments for SRMs in a multicenter international cohort, differing from prior studies limited to single-center data. Moreover, the application of trifecta outcomes as a comprehensive measure of treatment quality, an approach rarely used in PTA research, advances our understanding of the optimal application of these techniques.

Our study has limitations that should be recognized when interpreting our findings. Firstly, the retrospective design has inherent biases that could undermine the accuracy of our results. Furthermore, its multicentric nature could imply dissimilarities in terms of surgical techniques and follow-up protocols, potentially resulting in discrepancies in outcomes. However, despite these constraints, our study presents a large multicenter cohort comparison of long-term oncologic outcomes between CRYO and RFA. Future research and prospective clinical trials are warranted to address the need for high-quality prospective data regarding the clinical effectiveness of PTA in treating SRMs. A recent feasibility study demonstrated the viability of a cohort-embedded randomized controlled trial comparing PTA and robot-assisted PN (38).

## CONCLUSIONS

CRA and RFA ablation both provide favorable and durable cancer control and preservation of renal function in the treatment of cT1a renal masses. While complication profiles between the two techniques vary slightly, their comparable long-term oncologic outcomes support their use as effective nephron-sparing alternatives for poor surgical candidates. Prospective studies are encouraged to further substantiate these findings and refine patient selection criteria.

## COMPLIANCE WITH ETHICAL STANDARDS

### Ethics approval and consents

This study was conducted in accordance with the Declaration of Helsinki on ethical principles for medical research involving human subjects. It obtained exempt status after being reviewed by the local Ethics Committee. All patients provided written informed consent for the inclusion of their data in the database and for their use for scientific research purposes.

## Data Availability

The database and the raw results of the data analysis are available upon reasonable request to the corresponding author.
